# Complete chloroplast genomes of all six *Hosta* species occurring in Korea: molecular structures, comparative, and phylogenetic analyses

**DOI:** 10.1186/s12864-019-6215-y

**Published:** 2019-11-09

**Authors:** Soo-Rang Lee, Kyeonghee Kim, Byoung-Yoon Lee, Chae Eun Lim

**Affiliations:** 1 Department of Biological Science, Texas Tech University, Lubbock, TX USA; 2 National Institute of Biological Resources, 42 Hwangyeong-ro, Seo-gu, Incheon, 22689 South Korea

**Keywords:** *Hosta*, Chloroplast genome, Repeats, Codon usage, Sequence divergence, Phylogeny

## Abstract

**Background:**

The genus *Hosta* is a group of economically appreciated perennial herbs consisting of approximately 25 species that is endemic to eastern Asia. Due to considerable morphological variability, the genus has been well recognized as a group with taxonomic problems. Chloroplast is a cytoplasmic organelle with its own genome, which is the most commonly used for phylogenetic and genetic diversity analyses for land plants. To understand the genomic architecture of *Hosta* chloroplasts and examine the level of nucleotide and size variation, we newly sequenced four (*H*. *clausa*, *H. jonesii*, *H. minor*, and *H. venusta*) and analyzed six *Hosta* species (including the four, *H. capitata* and *H*. *yingeri*) distributed throughout South Korea.

**Results:**

The average size of complete chloroplast genomes for the *Hosta* taxa was 156,642 bp with a maximum size difference of ~ 300 bp. The overall gene content and organization across the six *Hosta* were nearly identical with a few exceptions. There was a single tRNA gene deletion in *H. jonesii* and four genes were pseudogenized in three taxa (*H. capitata*, *H. minor*, and *H. jonesii*). We did not find major structural variation, but there were a minor expansion and contractions in IR region for three species (*H. capitata*, *H. minor*, and *H. venusta)*. Sequence variations were higher in non-coding regions than in coding regions. Four genic and intergenic regions including two coding genes (*psbA* and *ndhD*) exhibited the largest sequence divergence showing potential as phylogenetic markers. We found compositional codon usage bias toward A/T at the third position. The *Hosta* plastomes had a comparable number of dispersed and tandem repeats (simple sequence repeats) to the ones identified in other angiosperm taxa. The phylogeny of 20 Agavoideae (Asparagaceae) taxa including the six *Hosta* species inferred from complete plastome data showed well resolved monophyletic clades for closely related taxa with high node supports.

**Conclusions:**

Our study provides detailed information on the chloroplast genome of the *Hosta* taxa. We identified nucleotide diversity hotspots and characterized types of repeats, which can be used for developing molecular markers applicable in various research area.

## Background

The genus *Hosta* Tratt. (Asparagaceae) is a group of economically important perennial herbs and distributed exclusively in eastern Asia [1–3]. As the plants have showy flowers and foliage, many *Hosta* species and the cultivars (~ 2500) are heavily exploited for gardening throughout all temperate regions [4]. The plants in *Hosta* are commonly called as plantain lily (bibichu in Korean) and have grown the popularity in gardens due to the advantages in cultivating due to the tolerance to shade and high soil moisture contents [5, 6]. Coupled with the horticultural importance, *Hosta* species provide critical values in medical areas. Recent studies revealed that the species are rich in saponins and amaryllidaceae alkaloids that are inhibiting tumor related and inflammatory activities [7, 8]. The *Hosta* plants also have been used as a folk medicine for treating multiple symptoms including multiple inflammatory diseases such as urethritis and pharyngolaryngitis in China and Japan [8].

The genus *Hosta* is placed in the family Asparagaceae since it was moved to the family from Liliaceae in the 1930s based on the cytological characteristics (2n = 60) [5]. There are approximately 22–25 species in the genus [1, 4], although the number of species (43 in Schmid) [5] and the relationships among the taxa have been problematic due to the extensive variability in morphology. The challenges in taxonomy of *Hosta* are also attributed to the confusions brought from the abundance of cultivars (number of cultivars reported > 2500) [2, 4]. The taxonomic difficulties are further complicated by the dearth of diagnostic characters as well as lack of comparative investigations on taxonomic keys between the dried herbarium specimens and the living plants from natural populations across varying environments [9]. In Korea, approximately 14 *Hosta* (11 species, 2 varieties, 1 cultivar) taxa have been reported thus far, however the number of species varies from 5 to 11 depending on the scholars working on the genus [10].

Organization of CP genomes are conserved throughout higher plants at the structural and genic level [11, 12]. Generally, in nearly all land plants, CP genomes are consisting of a single circular DNA molecule [11] and show quadripartite structure, i.e. a large single-copy region (LSC) and a small single-copy region (SSC) separated by inverted repeats (IRs). Although the extent of variation is not very large across flowering plants, the genome sizes of chloroplasts differ between species ranging from 107 kb (*Cathaya argyrophylla*) to 280 kb (*Pelargonium*) [11, 12]. There are approximately 120 to 130 genes in chloroplast genomes contributing to photosynthesis, transcription and translation [12]. The CP genomes are usually transmitted from one of the parents (supposedly no recombination occurring), mostly the mother in angiosperms [13]. The sequences of the CP genomes are conserved among taxa, thus the genomes often provide robust markers for phylogenetic analysis and divergence time estimation particularly at a higher taxonomic level [14].

Over a dozen of regions within the CP genome e.g. *ndh*F, *mat*K, and *trn*S*-trn*G have been widely amplified for the purpose of species identification, barcoding and phylogenies [15, 16]. Certainly, there is no universal region of CP genome that works best for all plant taxa. Also, despite the wide utilities of CP markers for taxonomic studies, the taxonomy of the most closely related taxa based on those markers often remains unresolved in many taxa due to the limited variation [15]. With the advent of next generation sequencing (NGS) technology, sequencing the whole CP genomes (plastome) for multiple taxa is feasible at a low cost. Recently the complete plastome sequences have been applied to reconstruct phylogenies on problematic taxa and has successfully resolved the enigmatic relationships [14, 17, 18]. Currently, four *Hosta* plastomes have been sequenced and two of those are publicly available in NCBI Organelle Genome Resources (http:// www.ncbi.nlm.nih.gov/genomes) [3, 19, 20]. In this study, we investigated the plastomes of all six Korean *Hosta* summarized by Chung and Kim [2]. We newly sequenced and assembled the whole plastomes of four species (*H*. *clausa*, *H. jonesii*, *H. minor*, and *H. venusta*). The plastome of *H*. *yingeri* (MF990205.1) [19] and *H. capitata* (MH581151) [20] were downloaded and added to the comparative analysis. The aims of our study were: 1) to determine the complete structure of plastomes for the four Korean *Hosta* species; 2) to compare sequence variation and molecular evolution among the six Korean *Hosta*; 3) to infer the phylogenetic relationship among the six Korean *Hosta* and reconstruct the phylogeny of the six species within the subfamily Agavoideae.

## Results

### Chloroplast genome assembly

**Fig. 1:**
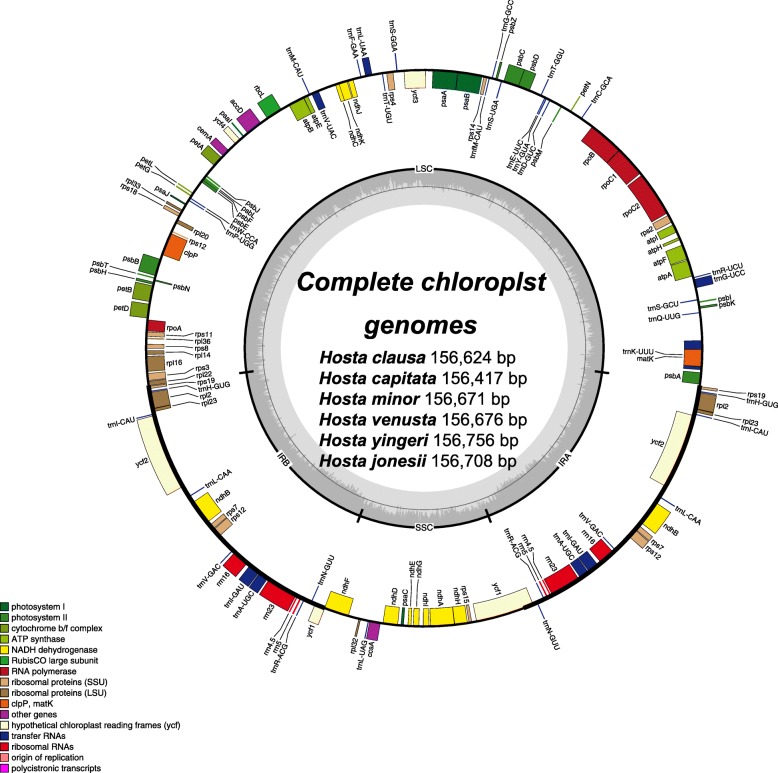
Chloroplast map of six *Hosta* species in Korea. The colored boxes represent conserved chloroplast genes. Genes shown inside the circle are transcribed clockwise, whereas genes outside the circle are transcribed counter-clockwise. The small grey bar graphs inner circle shows the GC contents

The genomic libraries from the four Korean *Hosta* species sequenced in our study produced ~ 7.8 to 13GB. The average number of reads after quality-based trimming was about 10 million and the mean coverage of the four plastome sequences is ~ 222 (Table 1). The percent of GC content did not vary much across the four plastome sequences and the average was 37.8% (Table 1). The complete CP genome size of the four *Hosta* ranged from 156,624 bp (*H*. *clausa*) to 156,708 bp (*H. jonesii*). As shown in most CP genomes, the four *Hosta* assembled in the study exhibited the typical quadripartite structure comprising of the four regions, a pair of inverted repeats (IRs 26,676–26,698 bp), LSC (85,004–85,099 bp) and SSC (18,225–18,244 bp; Fig. 1; Table 1).

**Table 1:** Sample information and summary of chloroplast genome characteristics for four *Hosta* species in Korea. The species acronyms are as followings: CLA- *H*. *clausa*; MIN- *H. minor*; VEN- *H. venusta*; JON- *H. jonesii*

Category	CLA	MIN	VEN	JON
Collection site	Mt. Daeam, Gangwon-do	Mt. Gaejwa, Busan-si	Seoguipo, Jeju-do	Yanga-ri, Gyeongsangnam-do
Voucher No.	NIBR-VP 000063279	NIBR-VP 0000538762	NIBR-VP 0000632798	NIBR-VP 0000538843
NCBI accession No.	MK732315	MK732316	MK732314	MK732318
Reads after trimming	6,690,938	12,171,518	5,497,667	10,194,165
Mean coverage	258.7	246.7	278.1	166.9
Total length (bp)	156,624	156,671	156,676	156,708
LSC length (bp)	85,004	85,094	85,099	85,088
SSC length (bp)	18,228	18,225	18,225	18,244
IRa length (bp)	26,696	26,676	26,676	26,698
IRb length (bp)	26,696	26,676	26,676	26,698
Total GC content (%)	37.81	37.80	37.80	37.80
Total number of genes	132	132	132	131

### Chloroplast genome annotation

Including *H*. *yingeri* and *H. capitata* (the CP genome sequences were downloaded from GenBank), the four Korean *Hosta* plastomes contained 132 genes, which consisted of 78 protein coding genes, 31tRNA- and 4 rRNA-coding genes (Table 2). There was a single tRNA gene (*trnT*-*UGA*) deletion found in *H. jonesii* resulting in 137 genes with 30 tRNAs for the species. Except for the one tRNA gene, all remaining genes and the composition found in the *H. jonesii* plastome was identical to those of the other five species. Of 138 genes, 20 genes (all 4 rRNAs, 8 of tRNAs, 6 of ribosomal protein coding genes and 2 of the other genes) were duplicated and placed in the IR regions (Table 2). Fifteen genes including nine protein coding genes (*atpF, ndhA, ndhB, petB, petD, rpoC1, rpl2, rpl16, rps12*) and six tRNAs contained one intron while two genes (*clpP* and *ycf3*) contained two introns (Table 2). About 42% of plastome sequences of the six Korean *Hosta* species came out as the coding region encoding tRNAs, rRNAs, and proteins. We found four pseudogenes *infA*^ψ^, *ycf15*^ψ^, *rps16*^ψ^ and *rps11*^ψ^ in three species *H. capitata*, *H. minor* and *H. jonesii* (Table 2).

**Table 2:** List of genes within chloroplast genomes of six *Hosta* species in Korea. ×2 refers to genes duplicated in the IR regions

Category	Group of genes	Names of genes
Transcription & Translation	Ribosomal protein, LSU	*rpl33*, *rpl20*, *rpl36*, *rpl14*, *rpl16* (× 2)^a^, *rpl22*, *rpl2*(× 2)^a^, *rpl23*(× 2), *rpl32*
	Ribosomal protein, SSU	*rps16*, *rps2*, *rps14*, *rps4*, *rps18*, *rps12*(× 2)^a^, *rps11*, *rps8*, *rps3*, *rps19*(× 2), *rps7*(× 2), *rps15*
	RNA polymerase	*rpoC2*, *rpoC1*^a^, *rpoB*, *rpoA*
	Ribosomal RNAs	*rrn16*(×2), *rrn23*(× 2), *rrn4.5*(× 2), *rrn5*(× 2)
	Transfer RNAs	*trnL-UAA*^a^, *trnF-GAA*, *trnV-UAC*^a^, *trnM-CAU*, *trnW-CCA*, *trnP-UGG*, *trnH-GUG*(×2), *trnI-CAU*(× 2), *trnL-CAA*(× 2), *trnV-GAC*(× 2), *trnI-GAU*(× 2)^a^, *trnA-UGC*(× 2)^a^, *trnR-ACG*(× 2), *trnN-GUU*(× 2), *trnL-UAG*, *trnR-UCU*, *trnD-GUC*, *trnC-GCA*, *trnQ-UUG*, *trnE-UUC*, *trnG-UCC*, *trnK-UUU*^a^, *trnfM-CAU*, *trnS-GCU*, *trnS-UGA*, *trnS-GGA*, *trnT-GGU*, *trnT-UGA*, *trnY-GUA*, *trnG-GCC*^a^, *trnT-UGU*
Photosynthesis	Photosystem I	*psaB*, *psaA*, *psaI*, *psaJ*, *psaC*
	Photosystem II	*psbA*, *psbK*, *psbI*, *psbM*, *psbD*, *psbC*, *psbZ*, *psbJ*, *psbL*, *psbF*, *psbE*, *psbB*, *psbT*, *psbN*, *psbH*, *petN*
	NADH dehydrogenase	*ndhJ*, *ndhK*, *ndhC*, *ndhB*(×2)^a^, *ndhF*, *ndhD*, *ndhE*, *ndhG*, *ndhI*, *ndhA*^a^, *ndhH*
	Cytochrome b6/f complex	*petN*, *petA*, *petL*, *petG*, *petB*^a^, *petD*^a^
	ATP synthase	*atpA*, *atpF*^a^, *atpH*, *atpI*, *atpE*, *atpB*
	Rubisco large subunit	*rbcL*
ATP-dependent protease subunit P		*clpP*^a^
Other genes	Chloroplast envelope membrane protein	*cemA*
	Maturase	*matK*
	c-type	*ccsA*
	Subunit Acetyl- CoA-Carboxylate	*accD*
	Photosystem I assembly& stability	*ycf3*^b^, *ycf4*
Conserved ORFs		*ycf1*, *ycf2*(×2)
Pseudogenes		*infA*^ψ^ (MIN/CAP), *ycf15*^ψ^ (MIN/CAP), *rps16*^ψ^ (JON), *rps11*^ψ^ (JON)

Abbreviations: *LSU rRNA* Large subunit ribosomal ribonucleic acid, *SSU rRNA* Small subunit ribosomal ribonucleic acid

Gene marked with asterisks are the gene with a single (^a^) or double (^b^) introns. ^ψ^Pseudogenes are presented in the species indicated with parentheses. See Table 1 legend for the species acronyms

### Comparative chloroplast genome structure and polymorphism

**Fig. 2:**
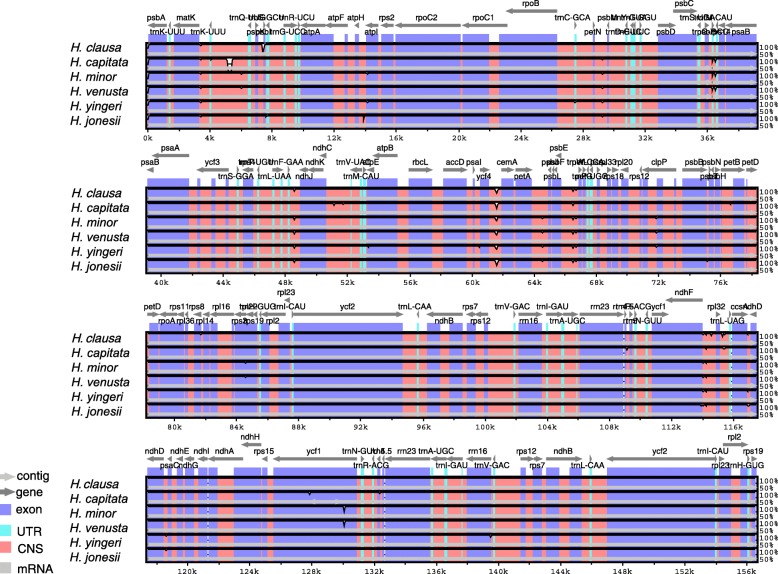
Plots of percent sequence identity of the chloroplast genomes of six Korean *Hosta* species with *H. ventricosa* (NCBI accession number: NC_032706.1) as a reference. The percentage of sequence identities were estimated and the plots were visualized in mVISTA

**Fig. 3:**
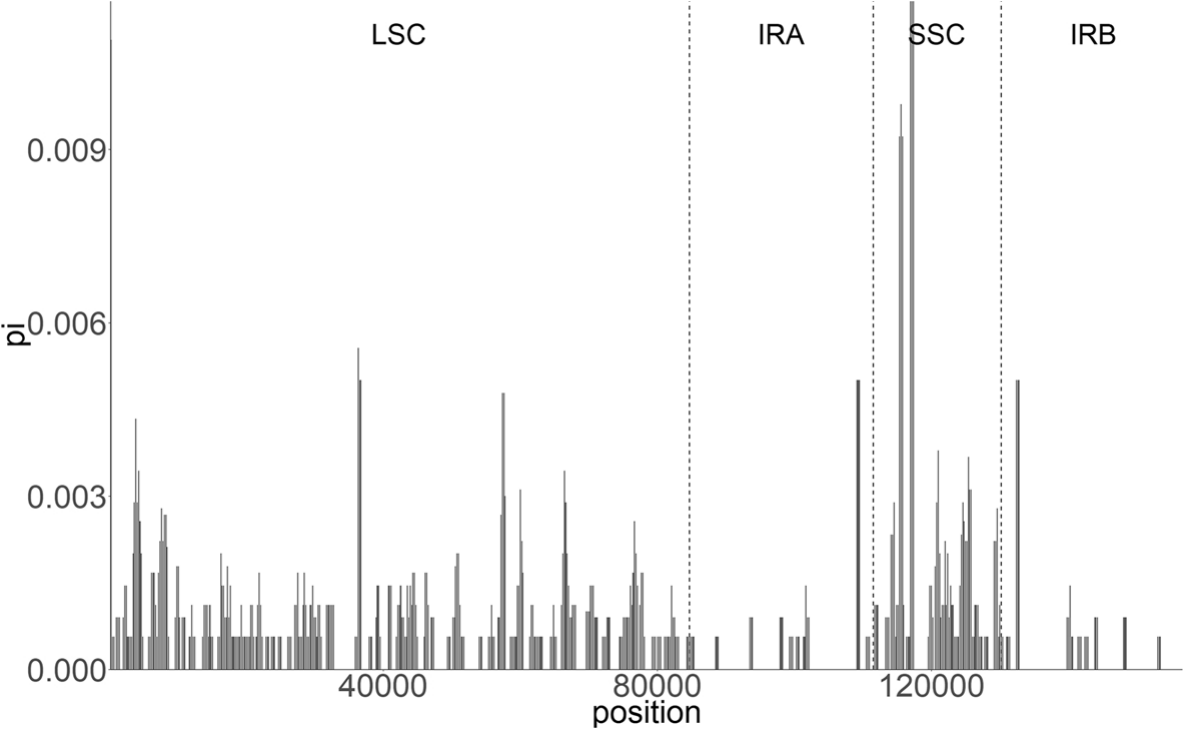
Plot of sliding window analysis on the whole chloroplast genome for nucleotide diversity (pi) compared among six *Hosta* species in Korea. The dashed lines are the borders of LSC, SSC and IR regions

The comparative sequence analysis of the six Korean *Hosta* revealed that the plastome sequences were fairly conserved across the six taxa with a few regions with variation (Fig. 2). Overall the sequences were more conserved in the coding regions, whereas, most of variation detected were found in non-coding sequence (CNS in Fig. 2) areas. The sequences of exons and UTRs were nearly identical throughout the six taxa except for *ycf1* for *H. capitata, H. minor* and *H. venusta* (Fig. 2). There was a slight variation detected on *rps19* for *H. minor* and *H. venusta*. We found the most projecting sequence polymorphism in *H. capitata* on the intergenic region between *trnK*-*UUU* and *trnQ*-*UUG* due to a 278 bp sequence deletion (Fig. 2). The amplicon size of *H. capitata* for the region was 231 bp, whereas the size of amplicons for the remaining five taxa was 509 bp (Additional file 1: Figure S1). The length difference between *H. capitata* and the other five *Hosta* taxa was 278 bp. We further examined sequence variability by computing nucleotide polymorphism (pi) among the six taxa. The average sequence diversity was 0.0007 and the pi ranged from 0 to 0.012 (Fig. 3). Overall the sequence diversities of IRs were more conserved (average pi = 0.0002) than the one calculated for LSC (average pi = 0.0008) and SSC region (average pi = 0.0016; Fig. 3). The average pi for non-coding region (0.0011) was higher than the one (0.0006) estimated for coding sequences. The most highly variable regions (pi > 0.05) include one tRNA (*trnL*-*UAG*: 0.012), two protein coding genes (*psbA*: 0.010, *ndhD*: 0.012) and one intergenic region (*ndhF*/*rpl32* IGS: 0.12). Based on the results of DNA sequence polymorphism we examined, the intra-specific polymorphisms were nearly zero except for *ndhD* gene in *H*. *clausa* (Additional file 1: Table S3 and Table S4). Overall, the *ndhD* gene showed the highest sequence polymorphism (pi = 0.01033), whereas the remaining three genes exhibited limited variation (Additional file 1: Table S3 and Table S4).

**Fig. 4:**
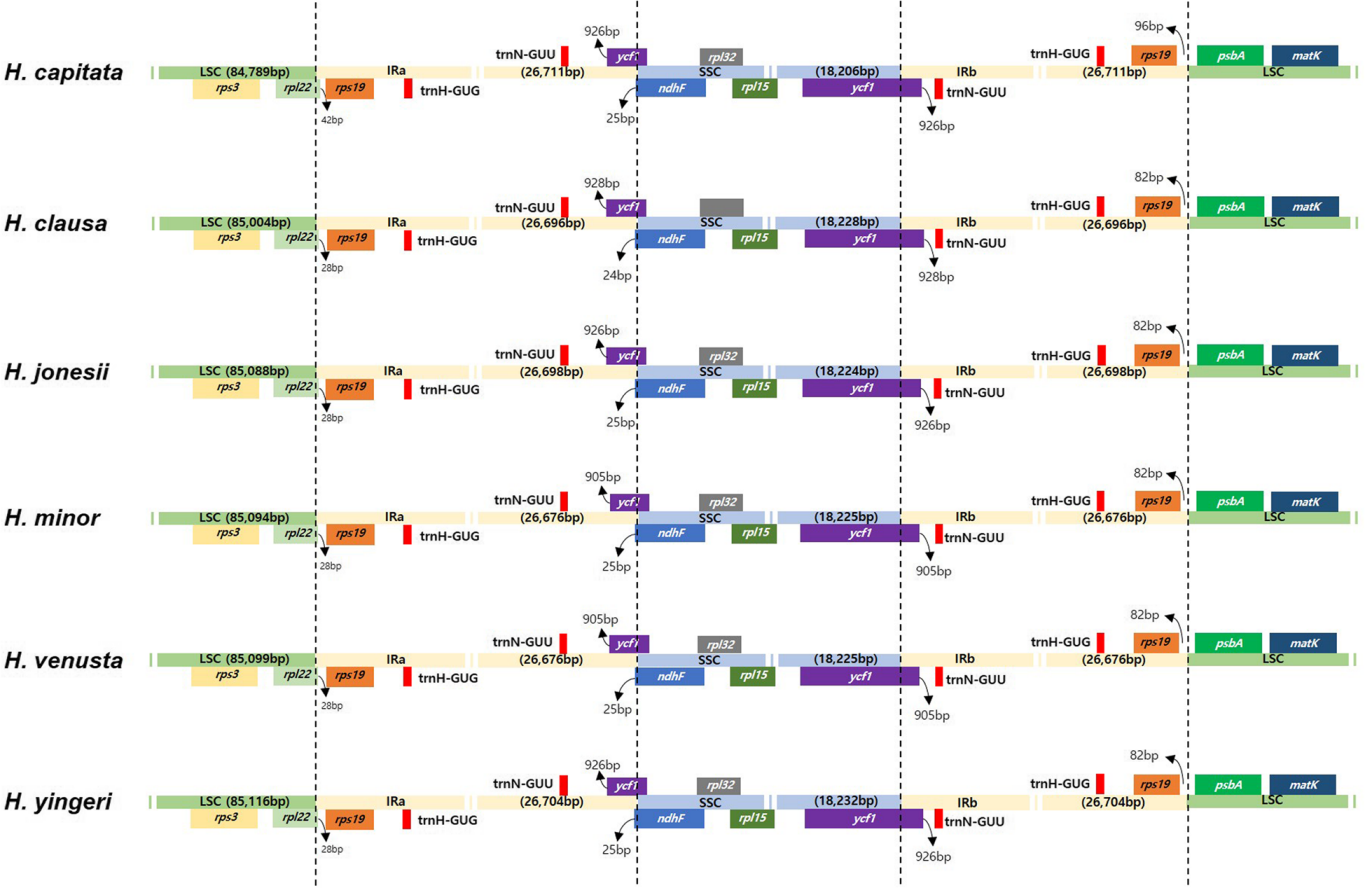
Comparisons of LSC, SSC and IR region boundaries among the chloroplast genomes of six Korean *Hosta* species

We compared the IR and SC boundaries of the six Korean *Hosta*. Overall, the organization of gene content and the size of genes shared high similarities among the six taxa although there were some distinctive variations. We found expansion and contraction of IR regions. The largest size of IR was shown in *H. capitata* despite the smallest overall plastome size (Table 1). Although the *rps19* genes of all six taxa were placed in the IR region, the location of the gene in *H. capitata* was the most distant from the boundary between the IR and LSC (Fig. 4). *rpl22* gene were positioned within the LSC with an 28 bp overlaps with the IRa for the five Korean *Hosta* species except for *H. capitata* (Fig. 4). The overlap was 14 bp longer in *H. capitata* indicating expansion of IR in the species. The border across IRb and SSC was placed in the region of *ycf1* gene with 926–928 bp tail section of the gene located in the IRb for most of the Korean *Hosta* (Fig. 4). However, the size of the tail section was reduced by ~ 20 bp length for *H. minor* and *H. venusta* suggesting contraction of the IR section in the two taxa (Fig. 4).

### Codon usage pattern

According to the codon usage analysis, overall 64 codons were present across of the six Korean *Hosta* species encoding 20 amino acids (AAs). Total number of codons for protein coding genes found was 26,505 in all six Korean *Hosta*. The effective number of codons were as followings: 3158 (*H*. *clausa*); 4002 (*H. capitata*); 4006 (*H. minor*); 5007 (*H. venusta*); 5018 (*H*. *yingeri*) and 4004 (*H. jonesii*). The most abundant AA among the 20 AAs was leucine (number of codons encoding leucine = 2735, 10.3%) followed by isoleucine (number of codons encoding isoleucine = 2287, 8.6%). Alanine was the least frequent AA in the Korean *Hosta*, which is encoded only by 309 codons (1.2%). The codon usage based on relative synonymous codon usage values (RSCU) did not vary among the six Korean *Hosta* species except for some decreases found in three AAs of *H. venusta* and *H. yingeri* (Additional file 1: Figure S2). Of the six *Hosta* species, *H. venusta* and *H. yingeri* had 47 codons more frequently used than the expected usage at equilibrium (RSCU > 1) while the rest of four *Hosta* species showed the codon usage bias (RSCU > 1) in 59 codons. All six *Hosta* had 59 codons less frequently used than the expected usage at equilibrium (RSCU < 1). Codons with A and/or U in the third position take up ~ 30% and ~ 24% of all codons respectively. The frequency of use for the start codons AUG and UGG, encoding methionine and tryptophan, showed no bias (RSCU = 1) in all Korean *Hosta* taxa.

### Tandem repeat and SSR

The total number of simple sequence repeats (SSRs) found in six Korean *Hosta* ranged from 51 to 59 (Table 3). Of these, the most abundant type of SSRs were the mono-nucleotide repeats with size of 10 to 16. Except for the mono-nucleotide SSR with C located in *ndhF* gene, nearly all mono repeat was composed of A or T in all six taxa. Over 60% of di-nucleotide SSRs were shown in the form of “AT” and the repeat number variation ranged from 10 to 18. We found four types of tetra-nucleotide SSRs in four of the six taxa, whereas *H. venusta* and *H. minor* had five different types of tetra-nucleotide SSRs (Table 3). There was no tri- and hexa- nucleotide SSRs in the six Korean *Hosta*. The type of compound SSRs differ across the six *Hosta* taxa. In addition to the SSR repeats, we further investigated the long repeats and identified 49 repeats consisting of on average 26 palindromic, 15 forward, 7 reverse and 1 complement repeats (Additional file 1: Table S1). The smallest unit size of the repeat was 18 while the largest unit size was 46. The majority of the repeats (ca. 88%) were size of less than 30 and nearly half of the repeats (ca. 47%) were situated in or at the border of genic regions. Among those repeats within the coding region, 4 palindromic and 5 forward repeats were located on *ycf2* (Additional file 1: Table S1).

**Table 3:** Distribution of simple sequence repeats (SSRs) in six *Hosta* species in Korea. c denotes for compound SSR of which comprised more than two SSRs adjacent to each other. The number of polymorphic SSRs were counted when the SSRs are polymorphic at least in one species

Number of SSRs (No. of polymorphic SSRs)								
Species	Unit size							Total
	1	2	3	4	5	6	c	
*Hosta clausa*	34 (11)	10 (1)	0	4	1	0	2 (1)	51 (12)
*Hosta capitata*	36 (14)	12 (4)	0	4	1 (1)	0	4 (3)	57 (22)
*Hosta minor*	35 (14)	10 (2)	0	5 (1)	1 (1)	0	2 (1)	53 (19)
*Hosta venusta*	36 (15)	10 (2)	0	5 (1)	1 (1)	0	1	53 (19)
*Hosta yingeri*	40 (19)	10 (2)	0	4	2 (2)	0	3 (2)	59 (25)
*Hosta jonesii*	39 (18)	10 (2)	0	4	2 (2)	0	2 (1)	57 (23)

### Phylogenetic inference

**Fig. 5:**
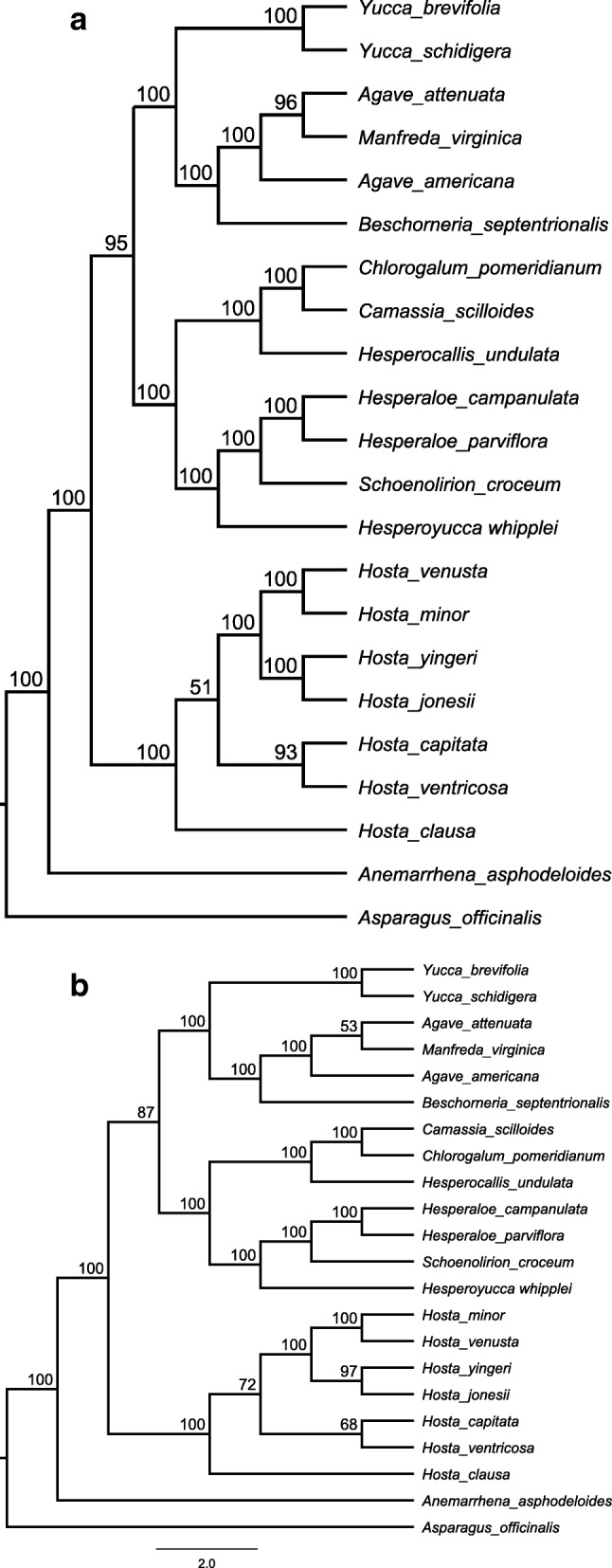
Phylogenetic relationships among the six *Hosta* species and 15 species in Agavoideae (Asparagaceae) inferred using Neighbor joining (NJ) and Maximum likelihood (ML) methods based on the whole chloroplast genomes. The values presented on each node are the bootstrap supports

We examined the phylogenetic relationships among 20 taxa in subfamily Agavoideae including the six Korean *Hosta* species using the whole plastome sequences. The overall topology of the phylogeny computed from both Maximum likelihood (ML) and Neighbor joining (NJ) was identical (Fig. 5). On average, the statistical supports for each node were fairly high except for a few tip nodes (Fig. 5). In the phylogeny, all seven *Hosta* taxa (see Table 1 and Additional file 1: Table S2 for the taxa names and GenBank accessions) formed a monophyletic group that is a sister to the group of most taxa in Agavoideae (Fig. 5). The genus *Anemarrhena* (*A*. *asphodeloides*) was positioned at the basal node. Among the seven *Hosta* taxa, *H. capitata* was the most closely related to *H. ventricosa* while *H. minor* formed another clade with *H. venusta* that is a sister to the clade of *H. jonesii* and *H. yingeri* (Fig. 5). *Hosta clausa* was place in the basal node in the monophyly of *Hosta* (Fig. 5).

## Discussion

Species in the genus *Hosta* are economically well recognized plants endemic to eastern Asia with taxonomic disputes due to the high morphological variabilities in Korea, China and Japan [1–3]. In the present study, we newly sequenced whole CP genomes for four Korean *Hosta* taxa and conducted comparative analyses on all six Korean *Hosta* CP genomes to understand the architecture of CP genomes in the taxa. We characterized gene organization along with codon usage pattern and found structural and size variations across the six *Hosta* taxa, which might be applicable for phylogenetic and population genetics studies.

Angiosperm plastomes have shown very little variation in size, structure and gene content [11, 12]. The *Hosta* plastomes that we analyzed revealed the typical quadripartite structure and fell in the expected size range (~ 15.7kbp) for angiosperm plants. Approximately 129 genes are present with 18 genes harboring introns across the angiosperm plastomes and the gene contents are also conserved [11, 21]. The gene annotation results in our study were consistent with the genetic properties of angiosperm plastomes. The number of genes found in CP genome from six Korean *Hosta* was ~ 130 and there were 18 genes with introns. The intron number is highly conserved throughout eudicots and most of monocots [21]. Our study found the same number of introns, 18, suggesting that the intron contents in *Hosta* are also similar to the ones from most of flowering plant clades. Although significant gene loss (> 30 genes) are observed in a small group of taxa (64 taxa), most of plant groups, only a handful of gene losses are detected [21]. It is believed that the most common gene losses in angiosperm, *infA* might have derived from transferring of the gene to the nucleus [22]. We found *infA* within two *Hosta* plastomes (*H. minor* and *H. capitata*), however the gene was pseudogenized by an internal stop codon.

Apart from a few exceptions, e.g. tobacco (171kbp) and geranium (217kbp), the plastome size variation is limited in angiosperm [11, 18]. The large size changes almost exclusively are accompanied by an elongation or deletion of inverted repeat regions, whereas most sequence variations are attributable to rather small length mutations mainly occurring in noncoding regions [11, 23]. In a recent comparative analysis of CP genomes across all land plants, monocots revealed a relatively high variation in size with an average plastome size of 14kbp [23]. The *Hosta* plastomes we analyzed showed a rather limited size variation (size difference < 85 bp) with one exception found in *H. capitata*. In the mVISTA result, there was 278 bp sequence deletion on *H. capitata* in the intergenic region around the *trnK*-*UUU* gene (Fig. 4). Our amplification result of the region indicates that the deletion is a unique feature of *H. capitata* (Additional file 1: Figure S1). The large length variations ranged from 50 to 1200 bp are not common in angiosperm plastomes [11]. The position of this large sequence deletion (around the border of LSC and IRb) coincides with the ones observed in angiosperms [11]. Although the causal mechanism for this large mutation is still elusive, it might offer valuable information on the evolution of plastome architecture as most of these variations shown in phylogenetic hotspots [11].

Besides the large length variation, we found sequence polymorphism in both genic and non-genic regions. Consistent with the diversity patterns found in most angiosperms [24–27], sequence divergence in non-coding regions (0.0011) was higher than the one in coding regions (0.0006). The overall nucleotide variability in *Hosta* plastomes was relatively lower than the ones found in other taxa (average pi = 0.009 in three *Papaver*; average pi = 0.003 in three *Cardiocrinum*) [25, 27]. Despite the lowered sequence variation, we identified four hyper-variable sites located in the SSC region (Fig. 3). We further examined the level of sequence polymorphism to determine whether these sites can be good candidates for a shallow level taxonomic studies i.e. inter- and intra-specific taxa in *Hosta* group. Notably, the results revealed very limited polymorphism for both inter- and intra-specific level. However, there was significantly high polymorphism found for *H*. *clausa* in *ndhD* gene. The number of variable sites among the two *H*. *clausa* samples from two different collection sites was 18, which is surprisingly high considering the limited number of variable sites (0–2) observed in the other genes and species (Additional file 1: Table S3 and Table S4). The highly inflated polymorphism may be in part due to long-term population isolation or the two samples might harbor different species or genetically distinct lineages. However, since our data set have limited sample size, the explanation must be taken with great caution. With the finding that our study discovered, some might further investigate diversity pattern of *ndhD* gene with larger sample size to determine the evolutionary history of the gene in the light of the species and population diversification.

It is hypothesized that the structural integrity of the whole plastomes is highly linked to the IR structure and the changes in plastome structure are often associated with IR expansions and contractions [28]. We investigated six Korean *Hosta* plastome structures and compared the sizes and the borders of the three components, LSC, SSC and IRs. Overall, our data suggests varying distribution of variations across the four plastome components with the least variation found in IRs (Figs. 3 & 4). The limited variation in IRs are largely consistent with the results of recent studies [25, 26]. However, we found IR expansions (*H. capitata*) and contractions (*H. minor* and *H. venusta*; Fig. 4). As the extent of expansions and contractions are small (< 20 bp), the IR structure changes doesn’t seem to significantly influence the whole plastome integrity.

Codon assignments for each of 20 amino acids are same across nearly all living organisms, yet the preference over individual codons largely differ among taxa [29]. Genome composition and selection towards increased translation efficiency are the two major factors affecting codon usage pattern [30, 31]. In the CP genome, the compositional bias associated with A/U rich positions is the primary cause of codon usage bias [32, 33]. The six *Hosta* CP genomes are low in GC content. In the six Korean *Hosta* taxa, we found a slight bias toward the nucleotide pair A/U. ~ 55% of total codons were with A/U at third position of the codons. However, the proportion of A/U at third position is significantly high for the biased codons with RSCU > 1. Among the codons with RSCU > 1 (more frequently used codons), over 76% had A/U at the third position.

On average, our plastome data found ~ 55 SSRs across the six *Hosta* taxa, which is slightly less than the ones reported in other angiosperm taxa (SSR numbers = 105 in *Betula*; 130 in *Paris*; 50 in *Chenopodium*; 250 in *Aconitum*; 48, in *Fagopyrum*) [24, 34–37]. We found inter-specific polymorphism in about 30 to 40% of the total SSRs (Table 3). Of the six *Hosta* taxa, *H. jonesii* harbored the highest number of SSRs that are polymorphic among species (Table 3). Simple sequence repeats, so called microsatellites are the tandem repeats that are most commonly used in population genetics studies due to the abundance, codominant mode of inheritance, and hyper-polymorphic nature [38]. The individual level of polymorphism may not be as high as the inter-specific polymorphism. However, the polymorphism we found only with a few species suggested that the SSRs we identified might be applicable for various population genetics studies on the *Hosta* taxa.

Aside from the two copies of inverted repeats, approximately 50 small repeats were dispersed throughout coding and non-coding regions of the six *Hosta* taxa. The repeat numbers are not significantly higher but comparable to the ones found in other angiosperms (dispersed repeat number in *Papaver* spp. = 49.; 21 in *Paris* spp.; 36 in *Passiflora*; 37 in *Aconitum*,) [24, 27, 36]. Repeats are highly correlated with the plastome rearrangement in various angiosperm taxa and can be a signature of recombination [39]. Repeats can provide recognition signals during recombination process as the repeated sequences have the potential to form secondary structures [40]. It has been believed that recombination rarely occurs in flowering plants due to the predominance of uniparental inheritance. However, evidence of intermolecular homologous recombination in flowering plants have been mounting [41, 42]. There was no record of plastome recombination in Asparagaceae, however plastome studies examining the recombination in the taxa are completely lacking thus far. Given higher number of repeats observed in our *Hosta* data, inter- and intra-specific plastome recombination might not be unlikely.

The genus *Hosta* have gained notorious recognition by the taxonomic confusion among the taxa due to morphological similarities, high variability of taxonomic characteristics and copious forms of cultivars [2, 4]. The taxonomic studies for *Hosta* taxa have been conducted mostly on pollen, flower and leaf morphology and a few molecular markers [9, 10], which may in part complicate the problems. Use of whole CP genome sequences has shown the considerable values to reconstruct the phylogenetic relationships among the complex taxa at various taxonomic levels [14, 18, 26]. We utilized the complete CP genome sequences of 21 taxa in subfamily Agavoideae (Asparagaceae) to infer phylogenetic relationships among the six Korean *Hosta* taxa and the related taxa. The plastome sequence of *Asparagus officinalis* (Asparagaceae) was assigned to an outgroup. There was no difference in the tree topology between the ML and NJ phylogenies with robust supports for the most clades suggesting a high confidence in the relationships among the clades and taxa (Fig. 5). The overall phylogenetic relationships among the 21 taxa computed from the complete plastome sequences (Fig. 5) were congruent to the one shown in the recent phylogenetic studies for family Asparagaceae [3, 43]. However, there was a slight conflict found on the relationships among the Korean *Hosta* taxa between our plastome based phylogeny and the phylogeny computed by 16 CP DNA restriction site mutations [9]. The latter put *H. yingeri* on a clade with *H. capitata*, whereas our plastome data support the clade of *H*. *yingeri* with *H. jonesii*. According to Chung et al. [2], *H*. *yingeri* showed more morphological similarities with *H. jonesii* than *H. capitata* by sharing the same smooth scape and spike-like inflorescence types. The high morphological similarity between *H*. *yingeri* and *H. jonesii* suggests that the complete plastome phylogeny might have a better resolution on those three species. These results suggest that the whole CP sequences provide a powerful tool for resolving specific level phylogeny.

## Conclusions

In conclusion, our study revealed the structural characteristics, distribution of sequence variation and repeats, gene content and organization for complete CP genomes in the six Korean *Hosta* species. Although structural variations are limited among the six *Hosta* plastomes, there were small IR region expansions and contractions in three taxa. We identified highly polymorphic regions of nucleotide variation that are potential molecular markers for phylogenetic studies. SSRs found in our plastome data might also provide intra-specific level polymorphic markers that can be used for population genetics studies. The increased number of dispersed repeats open to further evolutionary questions. Inter- and intra- specific recombination events might have happened in the past are likely be one plausible explanation for the increased number. Future studies might use the information of plastome architecture that we provided in this study and explore the characteristics of repeat elements.

## Methods

### Sampling, DNA isolation and sequencing

We collected fresh young leaf samples for four *Hosta* plants from four different localities listed in Table 1. The plants were identified based on the key morphological characters provided in Chung and Kim [2] and Jo and Kim [10]. The leaf samples were quickly dried with silica gel in a zip lock plastic bag upon the sampling and stored at room temperature until further use. We achieved all required permits for the protected areas from National Park Services and local governments. We prepared the voucher specimen for all four samples used and deposited them in the National Institute of Biological Resources with the accession numbers listed in Table 1.

Total Genomic DNA were extracted from each of the four *Hosta* plants using a DNeasy Plant Mini Kit (Qiagen Co., Hilden, Germany) following the manufacturer’s protocol. The extracted DNA were quantified in NanoDrop ND1000 (Thermo Fisher Scientific, Massachusetts, USA; quality cutoff, OD 260/280 ratio between 1.7–1.9) and visualized in a 1% agarose-gel electrophoresis for the quality check. Illumina paired-end (PE) libraries (read length: 2 × 125 bp) with insert sizes of 270 to 700 bp for each of the four *Hosta* species were constructed and sequenced on MiSeq platform (Illumina Inc., San Diego, CA) by Macrogen Inc. (http://www.macrogen.com/, Seoul, Korea). We removed poor quality reads (PHRED score of < 20) using the quality trim function implemented in CLC Assembly Cell package v. 4.2.1 (CLC Inc., Denmark).

### Genome assembly and annotation

We employed the low-coverage whole-genome sequence (dnaLCW) method [44] to assemble the complete CP genomes using both CLC de novo assembler in CLC Assembly Cell package and SOAPdenovo (SOAP package v. 1.12) with default parameters. Gaps were filled by the Gapcloser fuction in the SOAP package. To improve the CP genome assembly, we also conducted reference-based genome assembly using the CP genome sequences of *H. ventricosa* (GenBank accession = NC_032706.1). The contigs obtained from the primary de novo assemblies were aligned to the reference CP genome, then the aligned contigs were assembled to each chloroplast genome in Geneious v. 2019.0.4 (http://www.geneious.com).

We annotated the CP genomes assembled using the online tool, DOGMA (Dual Organellar GenoMe Annotator) [45] with a few adjustments for start and stop codons. Protein-coding genes were defined based on the plastid-bacterial genetic code. We also scanned all tRNAs with tRNAscan-SE [46] using the default settings to confirm the tRNA boundaries identified by DOGMA. The visual presentations of the plastome circular map were drawn in OGDRAW (http://ogdraw.mpimp-golm.mpg.de/). The annotated CP genome sequences of the four newly sequenced *Hosta* species in our study were then deposited in GenBank under the accession numbers listed in Table 1.

### Genome structure and comparative analysis

We compared the overall genome structure, genome size, gene content and repeats across all six Korean *Hosta* species including the CP genomes downloaded from GenBank (*H*. *yingeri* MF990205.1, *H. capitata* MH581151) [19]. The GC content was compared using Geneious. The whole plastome sequences of the six *Hosta* plants were aligned with MAFFT (http://mafft.cbrc.jp/alignment/server/) and visualized using Shuffle-LAGAN mode in mVISTA (http://genome.lbl.gov/vista/mvista/submit.shtml). For the mVISTA plot, we used the annotated CP genome of *H. ventricosa* as a reference. To determine whether 278 bp sequence deletion is a unique property of *H. capitata* or the result of sequencing error, we amplified the *trnK-UUU/trnQ-UUG* region, where the deletion is placed for the six *Hosta* species. The detailed method of amplification and data analysis are provided in the supplementary information (Additional file 1: S1). We also examined the sequence divergence among the six Korean *Hosta* species through a sliding window analysis computing pi among the chloroplast genomes in DnaSP v. 6.0 [47]. For the sequence divergence analysis, we applied the window size of 600 bp with a 200 bp step size. We further examined the level of polymorphism for the hyper-variable sites based on pi (*psbA*, *ndhD*, *trnL*, and *ndhF*-*rpl32* IGS). Two to three individuals were collected from different populations for the six Korean *Hosta* species (in total 13 individuals; Additional file 1: Table S3). We then extracted DNA from the 13 individuals and amplified the DNA using four primer pairs (Additional file 1: S2). The detailed conditions of amplification and the data analysis are provided in the supplementary information (Additional file 1: S2).

We found repeat elements using two approaches. Web-based simple sequence repeats finder MISA-web (https://webblast.ipk-gatersleben.de/misa/) was employed to identify SSRs with thresholds of 10 repeat units for mono-, 5 repeat units for di-, 4 repeat units for tri-, and 3 repeat units for tetra-, penta-, and hexa-nucleotide SSRs. Among the SSRs of each type, the polymorphic SSRs among the six species were counted by comparing the size of SSRs. We also investigated the size and type of repeats in the six Korean *Hosta* plastomes using REPuter [48]. For REPuter analysis, we set the parameters as follows: a minimal repeat size of 30 bp, hamming distance of 3 kb, and 90% or greater sequence identity. We analyzed codon usage to examine the distribution of codon usage using CodonW (http://codonw.sourceforge.net/) with RSCU ratio for all protein-coding genes.

### Phylogenetic analysis

We used the complete plastome sequences from all six Korean *Hosta* species with 14 plastome sequences of subfamily Agavoideae (Asparagaceae) obtained from GenBank including 1 *Hosta* species (*H. ventricosa*; genome size and the GenBank accession numbers are listed in Additional file 1: Table S2). *Asparagus officinalis* (Asparagaceae) was set as an outgroup for the phylogeny. The 21 plastome sequences including the outgroup were aligned using MAFFT and manually edited on Geneious alignment viewer. Gaps of sequences were treated as missing. We inferred the phylogeny using two approaches, a Neighbor joining and a Maximum likelihood analyses. The NJ phylogeny was performed according to Tamura-Nei distance [49] in Geneious Tree Builder. We constructed ML phylogeny using RAxML v. 8.2.4 with GTR GAMMA model with 1000 bootstrap replicates for evaluating the node support. To determine the best fitting substitution model, the Akaike information criteria (AIC) implemented in jModelTest v. 2.1.10 [50] was used.

## Supplementary information

**Additional file 1: Table S1.** The repeats shared by six *Hosta* species in Korea. The types are abbreviated as follows: F-forward, P-inverted (palindromic), C-complement, and R-reverse repeats. **Table S2.** The summary of the chloroplast genome sequences downloaded from GenBank for phylogenetic analysis. **Table S3.** Sample list used in the level of polymorphism test for the four hypervariable sites (*ndhF*-*rpl32* IGS, *ndhD*, *psbA*, and *trnL*). No. refers to number. Spp. indicates species. **Table S4.** Variable sites found in the six *Hosta* species in Korea. The species acronyms are as following: CAP- *H. capitata*; CLA- *H. clausa*; JON-*H. jonesii*; MIN- *H. minor*; VEN- *H. venusta*; YIN- *H. yingeri*. Dashes represent indels. **Figure S1.** Gel image of size variation in *trnK-UUU* ~ *trnQ-UUG* region amplified for the six Korean Hosta species. The size of the PCR fragments was determined by electrophoresis using the QIAxcel Advanced System and QIAxcel ScreenGel Software (Qiagen). The 5000 bp and 15 bp reference markers are marked in green. Lane A, *H. capitata*; lane B, *H. clausa*; lane C, *H. jonesii*; lane D, *H. minor*; lane E, *H. venusta*; lane F, *H. yingeri*. bp refers to the base pair. R indicates the reference marker. **Figure S2.** Codon contents for the optimal codons, i.e. codons occurring significantly more often in highly expressed genes, encoding 20 amino acids in the six *Hosta* chloroplast genomes in Korea. RSCU denotes for relative synonymous codon usage. **S1.** The method of amplification for LSC and IRb border to examine 278 bp deletion in *Hosta capitata*. **S2.** The method of amplification to identify intraspecific sequence polymorphism for the four hypervariable sites (*psbA*, *ndhD*, *trnL*, and *ndhF*-*rpl32* IGS) in the six Koran *Hosta* species.

## Data Availability

The four chloroplast genomes sequences we obtained from this study were archived in NCBI. The accession numbers are presented in Table 1.

## Abbreviations

AAs: Amino acids
AIC: Akaike information criteria
CNS: Non-coding sequence
CP: Chloroplasts
IRs: Inverted repeats
LSC: Large single-copy region
ML: Maximum likelihood
Nc: Effective number of codons
NGS: Next generation sequencing
NJ: Neighbor joining
Pi: Nucleotide polymorphism
plastome: Whole CP genomes
RSCU: Relative synonymous codon usage values
SSC: Small single-copy region
SSRs: Simple sequence repeats
