# Supplementary material for: Complete chloroplast genomes of all six Hosta species occurring in Korea: molecular structures, comparative, and phylogenetic analyses
Source: BMC Genomics. 2019 Nov 9;20:833. doi: 10.1186/s12864-019-6215-y (PMC6842461; doi:10.1186/s12864-019-6215-y)
Supplement: Supplementary file 1 — Additional file 1: Table S1. The repeats shared by six Hosta species in Korea. The types are abbreviated as follows: F-forward, P-inverted (palindromic), C-complement, and R-reverse repeats. Table S2. The summary of the chloroplast genome sequences downloaded from GenBank for phylogenetic analysis. Table S3. Sample list used in the level of polymorphism test for the four hypervariable sites (ndhF-rpl32 IGS, ndhD, psbA, and trnL). No. refers to number. Spp. indicates species. Table S4. Variable sites found in the six Hosta species in Korea. The species acronyms are as following: CAP- H. capitata; CLA- H. clausa; JON-H. jonesii; MIN- H. minor; VEN- H. venusta; YIN- H. yingeri. Dashes represent indels. Figure S1. Gel image of size variation in trnK-UUU ~ trnQ-UUG region amplified for the six Korean Hosta species. The size of the PCR fragments was determined by electrophoresis using the QIAxcel Advanced System and QIAxcel ScreenGel Software (Qiagen). The 5000 bp and 15 bp reference markers are marked in green. Lane A, H. capitata; lane B, H. clausa; lane C, H. jonesii; lane D, H. minor; lane E, H. venusta; lane F, H. yingeri. bp refers to the base pair. R indicates the reference marker. Figure S2. Codon contents for the optimal codons, i.e. codons occurring significantly more often in highly expressed genes, encoding 20 amino acids in the six Hosta chloroplast genomes in Korea. RSCU denotes for relative synonymous codon usage. S1. The method of amplification for LSC and IRb border to examine 278 bp deletion in Hosta capitata. S2. The method of amplification to identify intraspecific sequence polymorphism for the four hypervariable sites (psbA, ndhD, trnL, and ndhF-rpl32 IGS) in the six Koran Hosta species. [file 12864_2019_6215_MOESM1_ESM.docx]

Supplementary Information

Complete chloroplast genomes of all six *Hosta* species occurring in Korea: molecular structures, comparative, and phylogenetic analyses

Table S1. The repeats shared by six *Hosta* species in Korea. The types are abbreviated as follows: F-forward, P-inverted (palindromic), C-complement, and R-reverse repeats.

| Size | | | | | | Type | | | | | | Location |
| --- | --- | --- | --- | --- | --- | --- | --- | --- | --- | --- | --- | --- |
| CLA | CAP | MIN | VEN | YIN | JON | CLA | CAP | MIN | VEN | YIN | JON |  |
| 19 | 19 | 19 | 19 | 19 | 19 | P | P | P | P | P | P | *psbA* |
| 19 | 19 | 19 | 19 | 19 | 19 | F | F | F | F | F | F | *trnK-UUU* |
| 19 | 19 | 19 | 19 | 19 | 19 | P | P | P | P | P | P | *trnK-UUU* |
| 18 | 19 | 18 | 18 | 18 | 18 | R | F | C | C | C | C | IGS (*trnK-UUU*, *trnQ-UUG*) |
| 18 | 19 | 18 | 18 | 18 | 18 | P | R | P | P | P | P | IGS (*trnK-UUU*, *trnQ-UUG*) |
| 21 | 18 | 21 | 21 | 19 | 19 | R | P | R | R | C | R | IGS (*psbK*, *psbI*) |
| 25 | 21 | 25 | 25 | 21 | 21 | F | R | F | F | R | R | *trnS-GCU* |
| 24 | 25 | 24 | 24 | 25 | 25 | P | F | P | P | F | F | *trnS-GCU* |
| 22 | 24 | 30 | 30 | 24 | 24 | F | P | P | P | P | P | *trnG-UCC* |
| 18 | 30 | 22 | 22 | 22 | 22 | P | P | F | F | F | F | *atpF* |
| 18 | 22 | 18 | 18 | 18 | 18 | C | F | P | P | P | P | IGS *(atpF*, *atpH*) |
| 18 | 38 | 38 | 38 | 18 | 18 | P | P | P | P | C | C | *rpoC2* |
| 38 | 28 | 28 | 28 | 18 | 18 | P | P | P | P | P | P | IGS (*petN*, *psbM*) |
| 28 | 21 | 21 | 21 | 46 | 18 | P | P | P | P | P | P | IGS (*trnE-UUC*, *trnT-GGU*) |
| 21 | 21 | 21 | 21 | 28 | 38 | P | P | P | P | P | P | IGS (*trnE-UUC*, *trnT-GGU*) |
| 21 | 25 | 25 | 25 | 21 | 28 | P | R | R | R | P | P | *trnS-UGA* |
| 25 | 22 | 22 | 22 | 21 | 21 | R | P | P | P | P | P | IGS (*trnS-UGA*, *psbZ*) |
| 22 | 21 | 21 | 21 | 25 | 21 | P | F | F | F | R | P | *trnG-GCC* |
| 22 | 28 | 28 | 28 | 22 | 25 | P | P | P | P | P | R | IGS (*trnG-GCC*, *trnfM-CAU*) |
| 21 | 22 | 22 | 22 | 21 | 22 | F | P | P | P | F | P | *trnfM-CAU* |
| 28 | 41 | 41 | 41 | 28 | 21 | P | F | F | F | P | F | IGS (*trnfM-CAU*, *rps14*) |
| 22 | 20 | 20 | 20 | 22 | 28 | P | F | F | F | P | P | IGS (*rps14*, *psaB*) |
| 41 | 20 | 20 | 20 | 41 | 22 | F | P | P | P | F | P | *psaB* |
| 20 | 19 | 19 | 19 | 20 | 41 | F | F | F | F | F | F | IGS (*trnT-UGU*, *trnL-UAA*) |
| 20 | 19 | 19 | 19 | 20 | 20 | P | P | P | P | P | F | IGS (*trnT-UGU*, *trnL-UAA*) |
| 19 | 20 | 20 | 20 | 19 | 20 | F | R | R | R | F | P | *trnF-GAA* |
| 19 | 20 | 19 | 19 | 19 | 19 | P | P | R | R | P | F | *trnF-GAA* |
| 20 | 19 | 22 | 22 | 20 | 19 | R | R | F | F | R | P | *ndhC* |
| 19 | 20 | 20 | 20 | 19 | 20 | R | R | R | R | R | R | IGS (*accD*, *psaI*) |
| 22 | 20 | 19 | 19 | 22 | 19 | F | P | P | P | F | R | IGS (*ycf4*, *cemA*) |
| 19 | 19 | 20 | 20 | 19 | 19 | R | F | F | F | R | R | IGS (*psbE*, *petL*) |
| 19 | 19 | 20 | 20 | 19 | 20 | F | P | P | P | F | F | IGS (*rps3*, *rpl22*) |
| 19 | 25 | 19 | 19 | 19 | 20 | P | F | C | C | P | P | IGS (*rps3*, *rpl22*) |
| 25 | 25 | 25 | 25 | 25 | 25 | F | P | F | F | F | F | IGS (*trnI-CAU*, *ycf2*) |
| 25 | 25 | 25 | 25 | 25 | 25 | P | P | P | P | P | P | IGS (*trnI-CAU*, *ycf2*) |
| 25 | 30 | 25 | 25 | 25 | 25 | P | F | P | P | P | P | IGS (*trnI-CAU*, *ycf2*) |
| 30 | 30 | 30 | 30 | 30 | 30 | F | P | F | F | F | F | *ycf2* |
| 30 | 30 | 30 | 30 | 30 | 30 | P | P | P | P | P | P | *ycf2* |
| 30 | 22 | 30 | 30 | 30 | 30 | P | F | P | P | P | P | *ycf2* |
| 22 | 22 | 22 | 22 | 22 | 22 | F | P | F | F | F | F | *ycf2* |
| 22 | 22 | 22 | 22 | 22 | 22 | P | P | P | P | P | P | *ycf2* |
| 22 | 25 | 22 | 22 | 22 | 22 | P | P | P | P | P | P | *ycf2* |
| 25 | 19 | 25 | 25 | 25 | 25 | P | R | P | P | P | P | IGS (*rpl32*, *trnL-UAG*) |
| 24 | 24 | 24 | 24 | 24 | 24 | P | P | P | P | P | P | IGS (*ccsA*, *ndhD*) |
| 20 | 20 | 20 | 20 | 20 | 20 | P | P | P | P | P | P | *ycf1* |
| 19 | 19 | 19 | 19 | 19 | 19 | R | R | R | R | R | R | *ycf1* |
| 22 | 22 | 22 | 22 | 22 | 22 | F | F | F | F | F | F | *ycf2* |
| 30 | 30 | 30 | 30 | 30 | 30 | F | F | F | F | F | F | *ycf2* |
| 25 | 25 | 25 | 25 | 25 | 25 | F | F | F | F | F | F | *ycf2* |

Table S2. The summary of the chloroplast genome sequences downloaded from GenBank for phylogenetic analysis.

| Species | Genome size (bp) | Accession No. |
| --- | --- | --- |
| *Agave attenuata* | 157451 | NC_032696.1 |
| *Manfreda virginica* | 157308 | NC_032707.1 |
| *Agave americana* | 157274 | NC_032053.1 |
| *Beschorneria septentrionalis* | 157043 | NC_032699.1 |
| *Yucca brevifolia* | 158008 | NC_032711.1 |
| *Yucca schidigera* | 156158 | NC_032714.1 |
| *Hesperaloe campanulata* | 157446 | NC_032702.1 |
| *Hesperaloe parviflora* | 157393 | NC_032703.1 |
| *Hesperoyucca whipplei* | 157832 | NC_032705.1 |
| *Hosta ventricosa* | 156577 | NC_032706.1 |
| *Schoenolirion croceum* | 156605 | NC_032710.1 |
| *Hesperocallis undulata* | 157143 | NC_032704.1 |
| *Chlorogalum pomeridianum* | 157288 | NC_032701.1 |
| *Camassia scilloides* | 155453 | NC_032700.1 |
| *Anemarrhena asphodeloides* | 156867 | NC_032698.1 |
| *Asparagus officinalis* | 156699 | NC_034777.1 |

Table S3. Sample list used in the level of polymorphism test for the four hypervariable sites (*ndhF*-*rpl32* IGS, *ndhD*, *psbA*, and *trnL*). No. refers to number. Spp. indicates species.

| No. | Collecting sites  (voucher No.) | Gene  (GenBank accession No.) | No. of variable sites  Within Sp. | | | |
| --- | --- | --- | --- | --- | --- | --- |
|  |  |  | *ndhF*/*rpl32* | *ndhD* | *psbA* | *trnL* |
| *H. capitata* | | | | | | |
| 1 | Gohal-ri, Jeongseon-gun, Gangwon-do (NIBRVP0000538716) | MN398228, MN398215,  MN398241, MN398254 | 0 | 0 | 0 | 0 |
| 2 | Eochi-ri, Gwangyang-si, Jeollanam-do (NIBRVP0000538747) | MN398229, MN398216,  MN398242, MN398255 |  |  |  |  |
| *H. clausa* | | | | | | |
| 1 | Dosan-myeon, Andong-si, Gyeongsangbuk-do (NIBRVP0000538786) | MN398230, MN398217,  MN398243, MN398256 | 1 | 18 | 0 | 0 |
| 2 | Mt. Soyo, Dongducheon-si, Gyeonggi-do (NIBRVP0000538972) | MN398231, MN398218, MN398244, MN398257 |  |  |  |  |
| *H. jonesii* | | | | | | |
| 1 | Yanga-ri, Namhae-gun, Gyeongsangnam-do (NIBRVP0000538843) | MN398232, MN398219, MN398245, MN398258 | 2 | 0 | 0 | 0 |
| 2 | Bonghwa-ri, Namhae-gun, Gyeongsangnam-do (NIBRVP0000538796) | MN398233, MN398220, MN398246, MN398259 |  |  |  |  |
| *H. minor* | | | | | | |
| 1 | Beopgi-ri, Yangsan-si, Gyeongsangnam-do (NIBRVP0000538704) | MN398234, MN398221, MN398247, MN398260 | 0 | 0 | 0 | 0 |
| 2 | Dongsam-dong, Yeongdo-gu, Busan (NIBRVP0000538765) | MN398235, MN398222, MN398248, MN398261 |  |  |  |  |
| *H. venusta* | | | | | | |
| 1 | Dongheungdong, Seogwipo-si, Jeju-do (NIBRVP0000538769) | MN398236, MN398223, MN398249, MN398262 | 0 | 0 | 0 | 0 |
| 2 | Aewol-eup, Jeju-si, Jeju-do (NIBRVP0000538770) | MN398237, MN398224, MN398250, MN398263 |  |  |  |  |
| *H. yingeri* | | | | | | |
| 1 | Heuksan Island, Jeollanam-do (NIBRVP0000752803) | MN398239, MN398226, MN398252, MN398265 | 0 | 0 | 0 | 0 |
| 2 | Heuksan Island, Jeollanam-do (NIBRVP0000752804) | MN398240, MN398227, MN398253, MN398266 |  |  |  |  |
| No. of variable sites among spp. (all six Korean *Hosta* spp.) | | | | | | |
| *ndhF-rpl32* 3, *ndhF-rpl32* 18, *ndhD* 3, *psbA* 0 | | | | | | |

|  | Variable nucleotide sites in alignment | | | | | | | | | | | | | | | | | | | | | | | | | |
| --- | --- | --- | --- | --- | --- | --- | --- | --- | --- | --- | --- | --- | --- | --- | --- | --- | --- | --- | --- | --- | --- | --- | --- | --- | --- | --- |
| Accession no. | *ndhF-rpl32*  IGS | | |  | *ndhD* | | | | | | | | | | | | | | | | | |  | *psbA* | | |
| Position of variation | 0  1  0 | 0  1  2 | 3  4  8 |  | 2  4  2 | 2  4  3 | 2  4  4 | 2  4  5 | 2  4  7 | 2  4  8 | 2  4  9 | 2  5  1 | 2  5  2 | 2  5  3 | 2  5  4 | 2  5  6 | 2  5  7 | 2  5  8 | 2  6  0 | 2  6  1 | 2  6  2 | 2  6  3 |  | 0  3  4 | 0  4  2 | 4  2  3 |
| CAP1 | **C** | **A** | **G** |  | **C** | **A** | **T** | **T** | **G** | **A** | **A** | **C** | **A** | **C** | **A** | **G** | **G** | **A** | **T** | **C** | **A** | **T** |  | **T** | **A** | **G** |
| CAP2 | **C** | **A** | **G** |  | **C** | **A** | **T** | **T** | **G** | **A** | **A** | **C** | **A** | **C** | **A** | **G** | **G** | **A** | **T** | **C** | **A** | **T** |  | **T** | **A** | **G** |
| CLA1 | **C** | **A** | **G** |  | **A** | **T** | **G** | **A** | **T** | **C** | **C** | **T** | **G** | **T** | **G** | **T** | **T** | **C** | **A** | **A** | **T** | **G** |  | **A** | **A** | **G** |
| CLA2 | **T** | **A** | **G** |  | **A** | **T** | **G** | **A** | **T** | **C** | **C** | **T** | **G** | **T** | **G** | **T** | **T** | **C** | **A** | **A** | **T** | **G** |  | **A** | **A** | **G** |
| JON1 | **C** | **A** | **A** |  | **A** | **T** | **G** | **A** | **T** | **C** | **C** | **T** | **G** | **-** | **-** | **-** | **-** | **-** | **-** | **-** | **T** | **G** |  | **T** | **G** | **A** |
| JON2 | **T** | **C** | **A** |  | **A** | **T** | **G** | **A** | **T** | **C** | **C** | **T** | **G** | **-** | **-** | **-** | **-** | **-** | **-** | **-** | **T** | **G** |  | **T** | **G** | **A** |
| MIN1 | **C** | **A** | **A** |  | **C** | **A** | **T** | **T** | **G** | **A** | **A** | **C** | **A** | **C** | **A** | **G** | **G** | **A** | **T** | **C** | **A** | **T** |  | **T** | **G** | **G** |
| MIN2 | **C** | **A** | **A** |  | **C** | **A** | **T** | **T** | **G** | **A** | **A** | **C** | **A** | **C** | **A** | **G** | **G** | **A** | **T** | **C** | **A** | **T** |  | **T** | **G** | **G** |
| VEN1 | **C** | **A** | **A** |  | **C** | **A** | **T** | **T** | **G** | **A** | **A** | **C** | **A** | **C** | **A** | **G** | **G** | **A** | **T** | **C** | **A** | **T** |  | **T** | **G** | **G** |
| VEN2 | **C** | **A** | **A** |  | **C** | **A** | **T** | **T** | **G** | **A** | **A** | **C** | **A** | **C** | **A** | **G** | **G** | **A** | **T** | **C** | **A** | **T** |  | **T** | **G** | **G** |
| YIN1 | **C** | **A** | **A** |  | **A** | **T** | **G** | **A** | **T** | **C** | **C** | **T** | **G** | **T** | **G** | **T** | **T** | **C** | **A** | **A** | **T** | **G** |  | **T** | **G** | **G** |
| YIN2 | **C** | **A** | **A** |  | **A** | **T** | **G** | **A** | **T** | **C** | **C** | **T** | **G** | **T** | **G** | **T** | **T** | **C** | **A** | **A** | **T** | **G** |  | **T** | **G** | **G** |
| YIN3 | **C** | **A** | **A** |  | **A** | **T** | **G** | **A** | **T** | **C** | **C** | **T** | **G** | **T** | **G** | **T** | **T** | **C** | **A** | **A** | **T** | **G** |  | **T** | **G** | **G** |

Table S4. Variable sites found in the six *Hosta* species in Korea. The species acronyms are as following: CAP- *H. capitata*; CLA- *H. clausa*; JON-*H. jonesii*; MIN- *H. minor*; VEN- H*. venusta*; YIN- *H. yingeri*. Dashes represent indels.


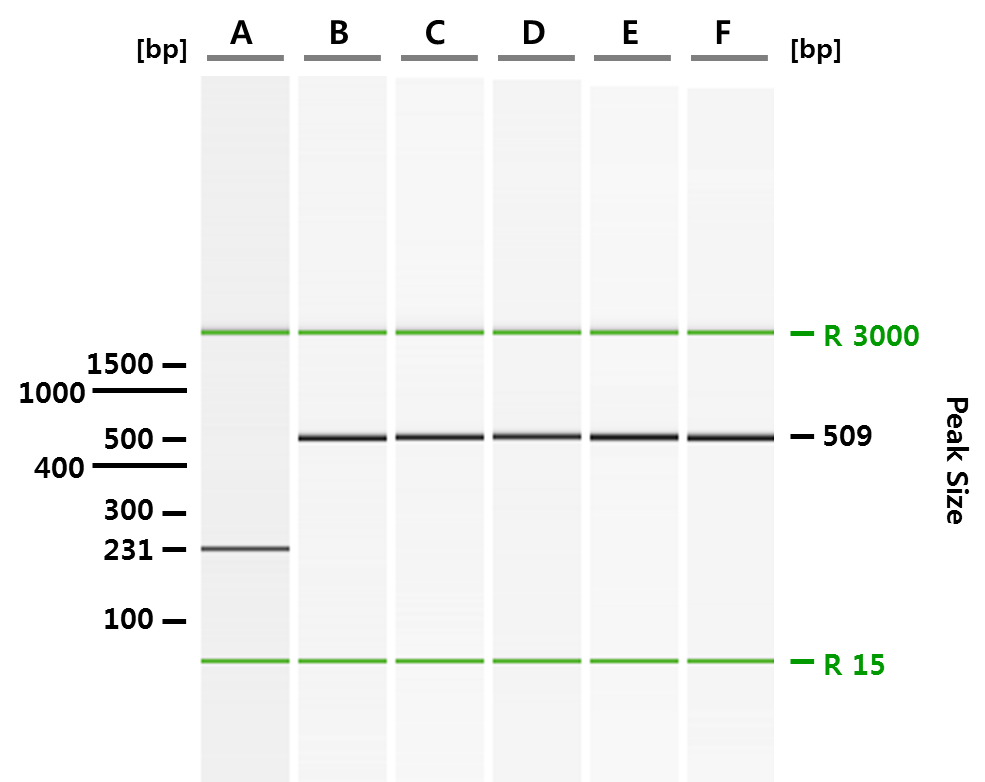


Figure S1. Gel image of size variation in *trnK-UUU* ~ *trnQ-UUG* region amplified for the six Korean Hosta species. The size of the PCR fragments was determined by electrophoresis using the QIAxcel Advanced System and QIAxcel ScreenGel Software (Qiagen). The 5,000 bp and 15 bp reference markers are marked in green. Lane A, *H. capitata*; lane B, *H. clausa*; lane C, *H. jonesii*; lane D, *H. minor*; lane E, *H. venusta*; lane F, *H. yingeri*. bp refers to the base pair. R indicates the reference marker.


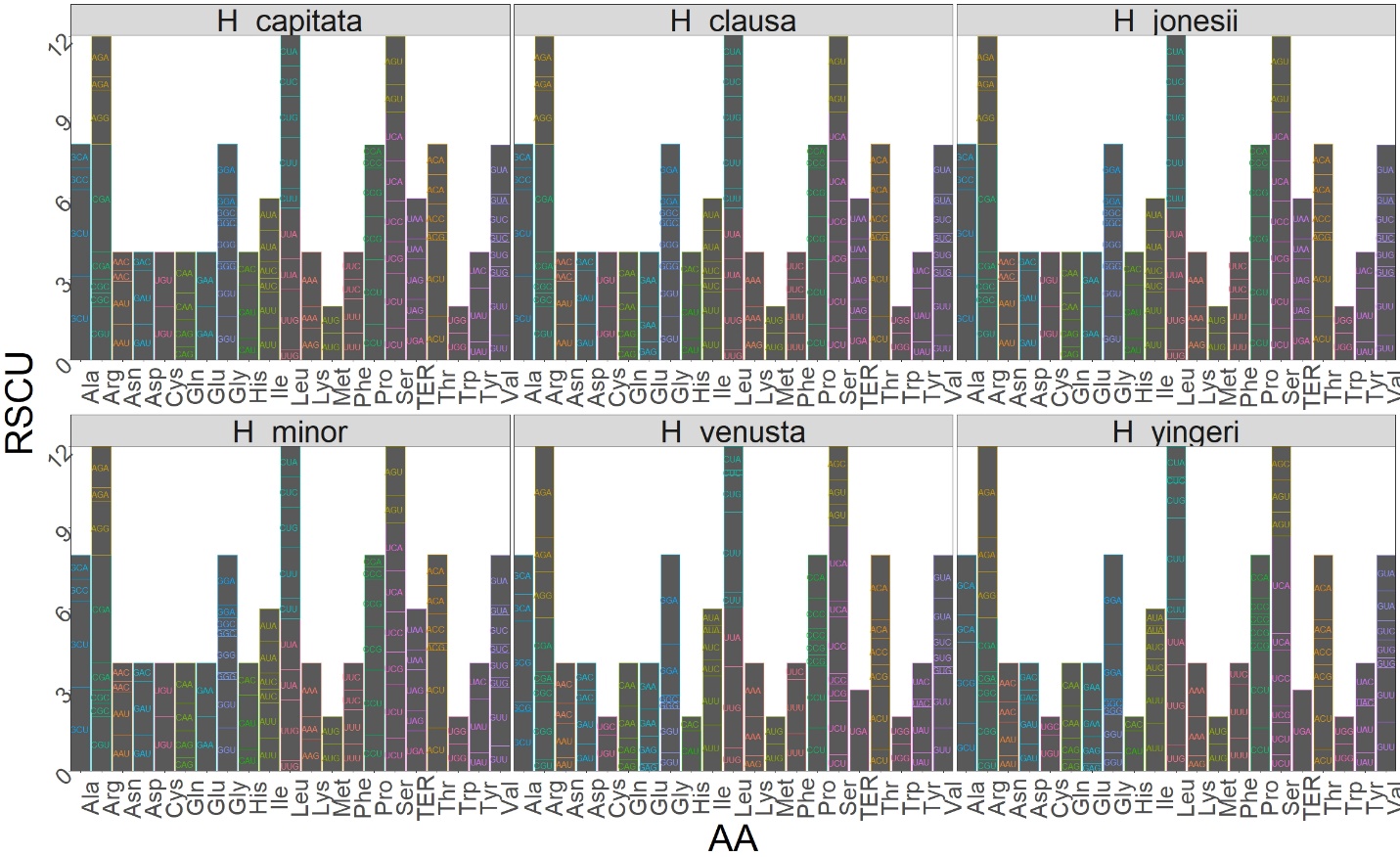


Figure S2. Codon contents for the optimal codons, i.e. codons occurring significantly more often in highly expressed genes, encoding 20 amino acids in the six *Hosta* chloroplast genomes in Korea. RSCU denotes for relative synonymous codon usage.

S1. The method of amplification for LSC and IRb border to examine 278bp deletion in *Hosta capitata*.

We developed a pair of primer set for the gene region, *trnK-UUU*/*trnQ-UUG*. The primer consists of 22 oligos for the forward direction (TGCTTTCTACCACATCGTTTCA) and 21 oligos for the reverse direction (TGAACCCTTGGTACTCGAAGA). For PCR reaction, 1㎕ of total DNA was included in a 20㎕ reaction mixture with commercialized PCR premix (iMOD PCR premix, SNC Co., Ltd., Korea). Polymerase Chain Reaction (PCR) was conducted using following program: 95℃ for 3 min, followed by 35 cycles of 95℃ for 60 s, 55℃ for 30 s, and 72℃ for 30 s, and finally 72℃ for 5 min. The sizes of amplicons for the six Korean *Hosta* species were visualized by Qiaxcel Advanced System (Qiagen, Genrmany) and the gel band images were made using QIAxcel Screengel Software program.

S2. The method of amplification to identify intraspecific sequence polymorphism for the four hypervariable sites (*psbA*, *ndhD*, *trnL*, and *ndhF*-*rpl32* IGS) in the six Koran *Hosta* species.

We developed 20-23bp forward and reverse primer sets for the four gene regions, *psbA* (F- TGGGTATAGCTCCCTCAACG and R-GTATGCGTCCTTGGATTGCT), *ndhD* (F-AATTGGGACCCCAAAGAAAC and R- GGGGAATAATTACTAGCCCAAAA), *trnL-UAA* (F- TGCAGAGACTCAATGGAAGC and R- TCCAAAATTTGCCCTAGTCC) and *ndhF*-*rpl32* IGS (F- TGACGGTCCAAGACCATAGA and R- CAATCAATCAGTTCCGCAAA). For each PCR reaction, 1㎕ of total DNA was included in a 20㎕ reaction mixture with commercialized PCR premix (iMOD PCR premix, SNC Co., Ltd., Korea). Polymerase Chain Reaction (PCR) was conducted using following program: 95℃ for 3 min, followed by 35 cycles of 95℃ for 60 s, 55℃ for 30 s, and 72℃ for 50 s, and finally 72℃ for 5 min. Sequence data were assembled and edited using Sequencer v. 5.1 (Gene Codes Co., USA), aligned with MAFFT, and manually inspected on Geneious alignment viewer implemented in Geneious v. 2019.0.4 (http://www.geneious.com). We calculated the number of variable sites for each gene region for both inter- and intra-specific level using DnaSP v. 6.0 [47]. We also computed pi among the six Korean *Hosta* species for the four amplified regions on DnaSP.
